# Sustainable water resources allocation for wetlands based on triple bottom line analytical hierarchy collaborative elicitation

**DOI:** 10.1007/s11356-024-35632-5

**Published:** 2024-11-28

**Authors:** Jorge Curiel-Esparza, Alberto Benitez-Navio, Manuel Martin-Utrillas, Jesus Martinez-Leon, Julian Canto-Perello

**Affiliations:** 1 Physical Technologies Center, Universitat Politecnica de Valencia, Camino de Vera S/N, 46022 Valencia, Spain; 2 Public Works Authority, Calle Fruela 6, 28011 Madrid, Spain; 3 The Jucar River Water Authority, Av Vicente Blasco Ibáñez 48, 46010 Valencia, Spain; 4 Department of Construction Engineering and Civil Engineering Projects, Universitat Politecnica de Valencia, 46022 Valencia, Spain

**Keywords:** Sustainable water allocation, Triple bottom line, Wetland conservation, Hybrid holistic index, Collaborative elicitation

## Abstract

Hydrological restoration of wetlands has become a critical pressing issue in environmental preservation due to climate change. This study seeks to develop a novel methodology to identify which type of water resources available are the most appropriate for restoring a particular wetland, considering a holistic perspective based on the triple bottom line (TBL) assessment, which is a logical framework for identifying and integrating social, environmental, and economic factors into decision-making processes. The elicitation was addressed through a comprehensive holistic index using analytic hierarchy process for ranking TBL dimensions and drivers. This new hybrid technique was applied for elaborating sustainable rules of water allocation to restore the wetlands of the Tablas de Daimiel National Park, located in central Spain. The environmental dimension was analyzed using six drivers: the synergistic use of infrastructures, the water resources location, the wastewater reuse, the energy consumption, the landscape degradation, and the impact on water resources. The social dimension was evaluated measuring three drivers: community acceptance, political acceptance, and market acceptance. And finally, the economic dimension was assessed through the expropriation of land costs, the infrastructure costs, the maintenance costs, and opportunity costs associated. These drivers guarantee traceability and transparency in the elicitation process, becoming a novel allocation framework to support policy makers in wetland conservation. Applying the proposed methodology, Tagus-Segura interbasin water transfer is the best ranked option (83.13%), closely followed by pumping well areas (79.12 and 78.24%) and wastewater recycling plants (74.34 and 68.26%). The unique holistic index proposed is a transparent and traceable decision support tool to address water allocation in wetland restoration.

## Introduction

In the World Water Development Report (UNESCO 2020), the Director-General of UNESCO, Audrey Azoulay, stated “Water does not need to be a problem, it can be part of the solution.” Unfortunately, the biodiversity of wetland ecosystems is under threat, posing significant challenges to sustainability (Mohibul et al. 2023; Hashim et al 2024). Wetlands play an important role in mitigating the greenhouse effect, especially peatlands and marshes, which are natural sinks for carbon dioxide (Mesene 2016). Despite their small footprint, wetlands play crucial roles, providing freshwater, habitats, storing 35% of organic carbon, and contributing to climate change mitigation (Mitsch and Gosselink 2015). Paris Agreement on Climate Change recognized the importance of the conservation and enhancement of sinks of the greenhouse gases and in its article 5.1 states that actions should be taken by the parties to the agreement. Climate change adaptation plans should be drawn up, focused on restoring wetlands to reduce the impacts of climate change on biodiversity and alleviate water scarcity (Fennessy and Lei 2018). Decline in wetlands is especially pronounced in regions suffering from water stress (Wang et al. 2023). Impact of climate change on wetlands will be more intense in these areas (Lefebvre et al. 2019; Doost et al 2024b). Wetland ecosystems depend on water levels and, consequently, climate and hydrology changes will have an impact on wetland sustainability, especially in arid regions under a climate change scenario (Wang and Qin 2017). Lefebvre et al. (2019) further showed that Spain is particularly threatened by water shortage due to climate changes. Hydrological restoration of wetlands involves supplying water from external sources such as nearby reservoirs, aquifers, wastewater treatment plants, or interbasin water transfers (Sanchez-Ramos et al. 2016; Xu et al. 2018; Arena et al. 2020). The water requirements of wetlands must be met through sustainable allocation of water resources facing social, environmental, and economic challenges (Yao et al. 2019). Water resources management may be qualified as sustainable if it addresses the current and future objectives of society, while maintaining ecological, environmental, and hydrological integrity.

Water is a scarce resource that supports economic growth, social development, and environmental protection. Assessing the long-term sustainability of large water resource systems under climate change is a complex issue because it involves evaluating social, environmental, and economic aspects (Haro-Monteagudo et al. 2020; Xu et al. 2023).

The objective of this paper is to develop a novel methodology for the decision-making process for the allocation of water resources in the hydrological restoration of wetlands needing water supply from external source for their preservation. Traditional decision-making in this field focuses mainly on tangible factors like cost and feasibility while often overlooking social and environmental impacts. Innovative studies addressing water resources allocation decision-making issue taking in consideration multiple criteria including social and environmental impacts focused in sustainability of water resources management has recently been published in fields like, e.g., the allocation of reservoirs for runoff management (Doost and Yaseen 2024), sustainable urban water management (Bessedik et al. 2023; Bouramdane 2023), water and environmental resources management (Motlaghzadeh et al. 2023), water resources allocation in river basins integrating efficiency, equity, and sustainability (Deng et al. 2022), sustainable water-agriculture-ecology management (Ma et al. 2023). A significant research gap exists in hydrological wetland restoration, particularly in considering the social and environmental impacts of external water supply. The aim of this paper is to fulfill this gap.

The triple bottom line (TBL) is a framework integrating the social, environmental, and economic drivers to achieve the UN Sustainable Development Goals (Chindasombatcharoen et al 2021). This approach ensures water resources are managed sustainably across environmental, social, and economic dimensions. In this research, TBL is applied to elicitate the most sustainable water resources for the restoration of a wetland. An optimal performance of the ecosystem requires a water resources management considering TBL to reach a compromise among environmental, social, and economic dimensions.

The methodology devised in this research has been applied to the management of water resources for the hydrological restoration of the Tablas de Daimiel National Park (TDNP), a 1900-ha wetland in central Spain designated Biosphere Reserve by the UNESCO in 1980 to propose the most sustainable options for the management of water resources, showing to be an efficient decision-making tool in the sustainable water resources allocation. This wetland is chosen as a representative wetland under water stress, with a peatland that plays a key role in reducing the greenhouse effect in need of hydrological restoration for its survival. The diversion of water to the TDNP wetland has an impact on vicinity water resources. These impacts highlight the need for careful planning and management of water diversion projects to balance human needs with environmental sustainability. The most sustainable water resources are the ones with the best TBL assessment. The water resources in a river basin constitute a global and very complex system, comprising ecological, economic, and social subsystems, which are interrelated (Meng et al. 2019). The uncertainty about the benefits and conflicting goals among available water resources presents a complex management framework. Once the hydrological restoration of the wetland is decided (Maleki, et al. 2018) and constitutes a priority against unsustainable agriculture or other unsustainable uses of water in arid regions, the criterion is to maximize and preserve the sustainability of water resources. The impact will also depend on the amount of water needed by the wetland (Liu et al. 2018). The sustainable selection of water resources to restore a wetland poses a management elicitation problem. Expert elicitation is the parameterization and synthesis of judgments through a scientific consensus technique from the panel of stakeholders and officials. Therefore, decisions should be undertaken based on a systematic and comprehensive procedure with enough consensus and transparency to avoid lack of acceptance.

A collaborative elicitation index for management of water resources in hydrological wetland restoration has been developed. The proposed hybrid allocation method uses analytical hierarchy process (AHP) for rating TBL dimensions and drivers. The AHP technique is based on paired comparison decision-making from panelists through a hierarchical structure of several levels (Saaty 2012). AHP can incorporate both tangible and intangible criteria by integrating both types of factors into a single framework and enabling a comprehensive evaluation of all relevant criteria (Martin-Utrillas et al. 2015; Amin et al. 2023). Additionally, the AHP rating method allows any number of water resources to be analyzed. Azarnivand et al. (2015) addressed the problem of water and environmental management of Lake Urmia, an endorheic salt lake in Iran. Sun et al. (2016) assessed the sustainability of regional water resources in the city of Bayannur. Karatayev et al. (2017) analyzed the priorities and challenges for a sustainable management of water resources in Kazakhstan. Canto-Perello et al. (2017) developed a hybrid model combining AHP and VIKOR techniques to achieve consensus in prioritizing river rehabilitation projects based on social, economic, and landscape drivers. Zhou et al. (2016) assessed water resources and sustainability in the Jinsha River basin. Kharabsheh et al. (2019) studied water resources management to help decision makers to address this problem in a sustainable and efficient way in arid regions. Sadek and Hagagg (2020) devised a groundwater sustainability index using AHP/GIS. Doost and Yaseen (2023) works was based on remote sensing imagery and water table data analyzed using an advanced GIS environment. In this research, the elicitation of water resources for hydrological restoration of a wetland is addressed through a comprehensive rating index using AHP for prioritizing TBL dimensions and drivers. The elicitation objective is to select the most sustainable water resources considering TBL. This novel hybrid technique allows prioritizing among the different available water resources to meet environmental, social, and economic dimensions.

## Methodology

### Study location

**Fig. 1:**
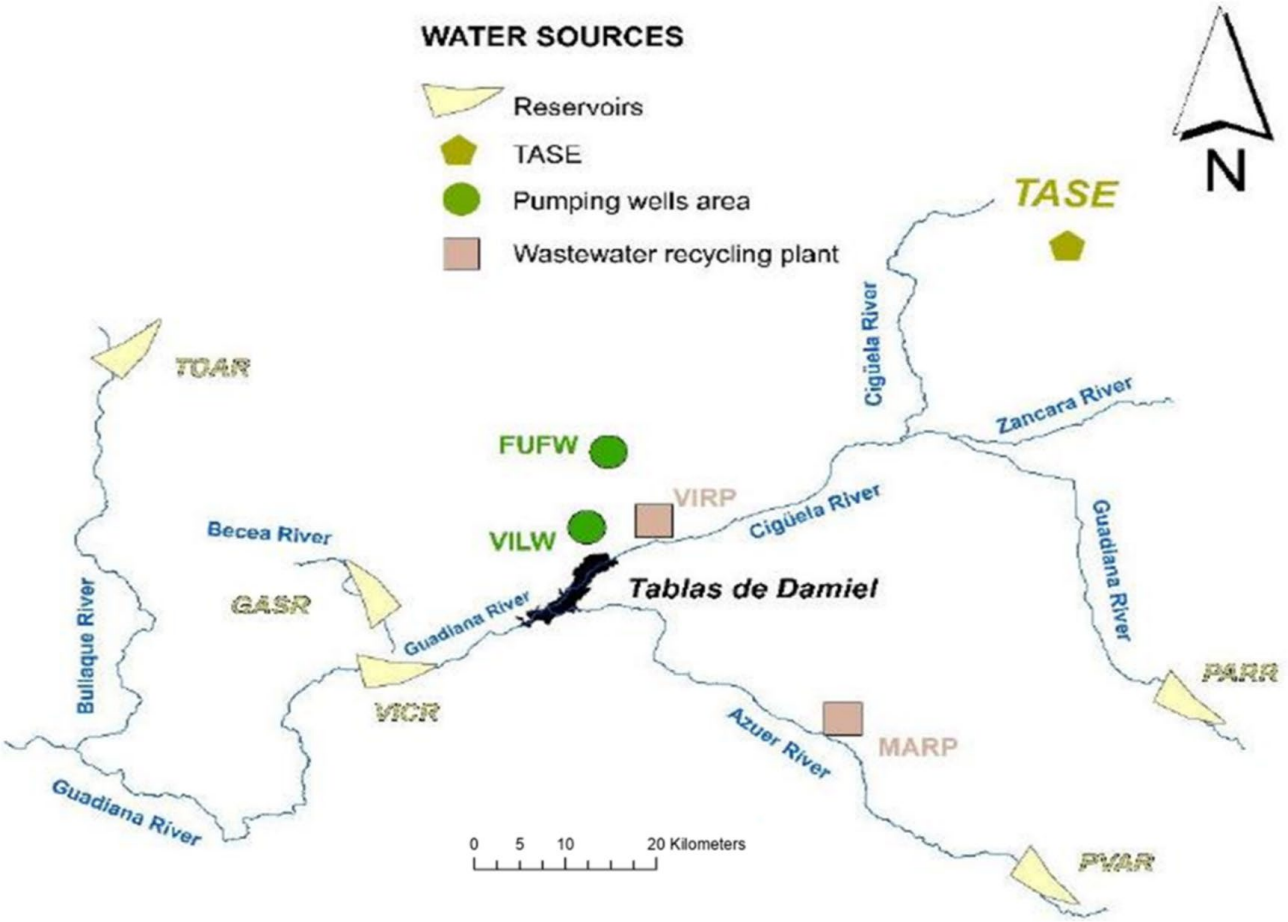
Tablas de Daimiel National Park, Upper and Lower Guadiana River Basin

The Tablas de Damiel National Park (TDNP) is a wetland in south central Spain that was declared a National Park by Spanish authorities in 1973 (BOE 1973). This Mediterranean alluvial plain is a threatened wetland in a semi-arid area. TDNP is located within La Mancha Húmeda, a region of wetlands and lagoons. As shown in Fig. 1, the crossing of the Guadiana River and its tributary Cigüela creates an alluvial plain in the great La Mancha plain, where the TDNP wetland constitutes a shallow pond of 1938 ha. In February 1981, UNESCO declared this area of 2500 km^2^ a Biosphere Reserve. In 1982, TDNP was also included in the Ramsar List of Wetlands of International Importance, and in 1987, under the Birds Directive of the European Union, it was declared a Special Protection Area for birds (Moreno 2019).

The water from the TDNP is a mixture between the influx of brackish water from the flooding of the Cigüela River and the underground outcrop of the Guadiana River aquifer (Castaño et al. 2008; Álvarez-Cobelas et al 2001). The latter is mainly formed by the discharge of the Western Mancha aquifer through a set of springs in the Ojos del Guadiana area (Castaño et al. 2012). Excessive withdrawals and frantic drilling of irrigation wells led to the overexploitation of the aquifer (Closas et al. 2017; Aguilera and Moreno 2019). In 1990, the wetland was added to the Montreux Record, as human activities have degraded TDNP ecosystems (Bravo-Martin et al. 2019). The main threat to the future existence of TDNP is water scarcity (Aguilera et al. 2013). This is a major hydrological problem aggravated by the effects of climate change and desertification (Sun et al. 2019; Doost et al. 2024a). The Mediterranean region is a desertification hotspot and the European area most threatened by this process (Prăvălie et al. 2017). In addition, TDNP has a peatland with high carbon content. These types of soils can self-ignite by starting smoldering peat fires (Restuccia et al. 2017), when the water content in the soil is below the tolerable minimum (Aguilera et al. 2016). In drought episodes, an external water inflow is mandatory to prevent the peat from self-ignite (Moreno et al. 2011). Water resources must be allocated to supply water to the wetland. Superficial water from reservoirs, aquifer groundwater, treated wastewater, and water from the Tagus-Segura interbasin transfer are possible resources (Canto-Perello et al. 2021).

### Elicitation hierarchy structure

The elicitation technique used to select water resources is based on the application of the analytic hierarchy process (AHP) with ratings through indicators (Saaty 2012). AHP allows analyzing the consistency of the experts’ responses. AHP calculates a consistency ratio by comparing the consistency index of the actual matrix with that of a random matrix. If the consistency ratio is close to 0, the judgments are considered consistent. The consistency analysis can be found in the following section. The allocation of water resources for the wetland restoration has an impact on the environment and human activities (Wang et al. 2018). This impact presents intangible factors that must be assessed. Therefore, selecting the most sustainable water resources poses a TBL decision-making problem (Curiel-Esparza et al. 2015a). AHP allows the inclusion of input from various stakeholders, including government agencies, local communities, and environmental groups. This guarantees that the decision-making process remains equitable and considers the needs and concerns of all stakeholders. The first step is the determination of the elicitation hierarchy structure for evaluating the water resources. A panel of nine experts was selected among stakeholders and officials. The recommended number of experts should be between eight and twelve on this type of panel (Alvarez Etxeberria et al. 2015). Selecting an expert panel of nine members is a strategic decision that balances diversity of expertise with manageability. This number of panelists allows for the inclusion of professionals from different fields, fostering a comprehensive evaluation process. Each panelist brings in-depth knowledge in their specific area, contributing to a well-rounded analysis. Through anonymous open-ended surveys, the panel has selected drivers based on TBL. The experts are allowed to reconsider their initial judgments (Curiel-Esparza et al. 2015b; Canto-Perello et al. 2018). Therefore, anonymized survey results are sent back to the panelists to reach consensus.

**Fig. 2:**
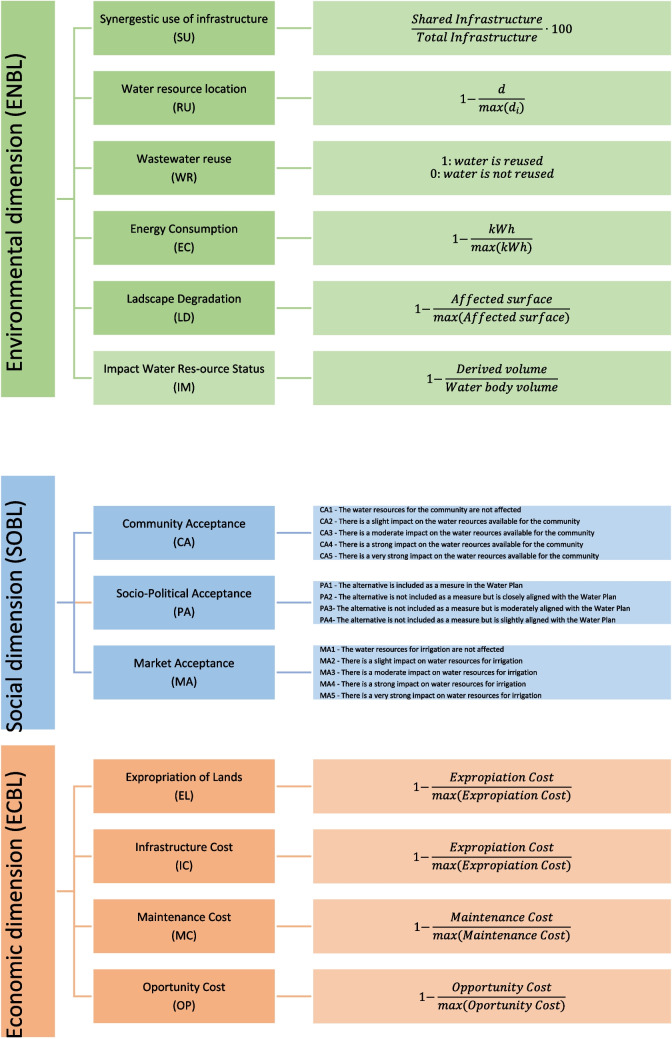
TDNP triple bottom line hierarchical elicitation structure including indicators and rating levels

The hierarchical elicitation structure, including drivers and ratings, is shown in Fig. 2. The first level in the elicitation hierarchy is the overall objective, the evaluation of available water resources for the wetland restoration. The TBL dimensions (environmental, social, and economic) are the second level and the drivers the third level. Six of them are related to the environmental dimension, three to the social dimension, and four to the economic dimension. Table 1 shows geographic coordinates and typologies of water resources under study to be assessed. These resources are identified in a previous study, and their location is displayed in Fig. 1 (Canto-Perello et al. 2021).

**Table 1:** Water resources under assessment for restoring the TDNP wetland

Water resource	Location	Type
Vicario reservoir (VICR)	39° 03′ 32″ N	Superficial water
	04° 00′ 10″ W	
Gasset reservoir (GASR)	39° 07′ 44″ N	Superficial water
	03° 56′ 15″ W	
Torre de Abraham reservoir (TOAR)	39° 24′ 20″ N	Superficial water
	04° 15′ 01″ W	
Puerto de Vallehermoso reservoir (PVAR)	38° 52′ 20″ N	Superficial water
	03° 10′ 00″ W	
Peñarroya reservoir (PARR)	38° 03′ 40″ N	Superficial water
	03° 16′ 30″ W	
Tagus-Segura interbasin water transfer (TASE)	39° 58′ 36″ N	Superficial water
	02° 44′ 12″ W	
Villarrubia pumping wells area (VILW)	39° 13′ 51″ N	Groundwater
	03° 36′ 56″ W	
Fuente Fresno pumping wells area (FUFW)	39° 14′ 20″ N	Groundwater
	03° 47′ 26″ W	
Manzanares wastewater recycling plant (MARP)	39° 00′ 15″ N	Treated wastewater
	03° 24′ 01″ W	
Villarrubia wastewater recycling plant (VIRP)	39° 12′ 12″ N	Treated wastewater
	03° 36′ 45″ W	

### Methodology for data collection, analysis and evaluation of water resources

Three TBL dimensions and thirteen drivers have been established, as shown in Fig. 2. Firstly, weights were assigned for each TBL dimension and driver applying AHP. A pairwise comparison technique consists of comparing two dimensions or drivers simultaneously. The 9-point Saaty scale is used to obtain experts’ judgments for dimensions and drivers. The individual judgments of the panelists are aggregated using the geometric mean method. The survey procedure leads to four reciprocal symmetric elicitation matrices: TBL dimension elicitation matrix (TBLEM), environmental dimension elicitation matrix (ENVEM), social dimension elicitation matrix (SOCEM), and economic dimension elicitation matrix (ECOEM). Denoting these matrices by $P=\left[{p}_{ij}\right]$, where ${p}_{ii}$ is equal to one because any element on the diagonal compares a dimension or a driver with itself. On the other hand, ${p}_{ij}$ is equal to $1/{p}_{ji}$ because the elements located in symmetric positions are reciprocal.

TBLEM is constructed with the priorities of the environmental, social, and economic dimensions, as shown in Table 2. And the other three elicitation matrices are obtained using the priorities of the environmental, as shown in Table 3, social, as shown in Table 4, and economic drivers, as shown in Table 5. To evaluate the priority of each individual dimension or driver, the eigenvector method has been applied to the four elicitation matrices. The dimension or driver priority vector (*PV*) is the main eigenvector of each elicitation matrix. The priority vector is obtained by solving a linear system:

$$\left[P\right]\cdot \left[PV\right]=\lambda \cdot \left[PV\right]$$

$$det\left(\left[P\right]-\lambda \left[I\right]\right)=0$$

where *λ* is the eigenvalue of the elicitation matrix, and [*I*] is the identity matrix. When using pairwise comparisons, errors coming out of inconsistency in judgments influence the final priority (Saaty 2012). In AHP, the consistency of the elicitation matrices can be measured by the consistency ratio (*CR*). To avoid inconsistencies, a maximum *CR* must be guaranteed. The maximum acceptable *CR* varies slightly depending on the order of the matrix. For a 3 × 3 matrix, the maximum acceptable *CR* is 0.05. In a 4 × 4 matrix, the maximum acceptable *CR* is 0.09. And finally, matrices of order 5 and above have the maximum acceptable *CR* in 0.10. These thresholds help ensure that the pairwise comparisons are consistent enough to produce reliable results. If the *CR* surpasses these thresholds, it suggests a notable level of inconsistency. The *CR* is obtained by the relationship between the consistency index (*CI*) and the random consistency index (*RCI*), as shown below:

**Table 2:** Priority vector and consistency analysis of TBLEM matrix assessing the environmental, social, and economic dimensions

	Environmental dimension	Social dimension	Economic dimension	Priority vector
Environmental dimension	1	1.3180	2.7938	0.4676
Social dimension	0.7587	1	2.4727	0.3734
Economic dimension	0.3579	0.4044	1	0.1590

*λ*_max_ = 3.0026; *CI* = 0.0013; *CR* = 0.0025 < 0.05 OK

**Table 3:** Priority vector and consistency analysis of ENVEM matrix assessing the environmental indicators

	*SU*	*RU*	*WR*	*EC*	*LD*	IM	Priority vector
SU	1	1.1298	0.4718	0.3338	0.3131	0.1717	0.0626
RU	0.8851	1	0.6504	0.3131	0.2941	0.2001	0.0643
WR	2.1197	1.5375	1	1.0000	0.5757	0.3975	0.1293
EC	2.9959	3.1936	1.0000	1	0.3992	0.3850	0.1481
LD	3.1936	3.4002	1.7371	2.5053	1	0.5576	0.2381
IM	5.8247	4.9974	2.5154	2.5971	1.7935	1	0.3576

*λ*_max_ = 6.0961; *CI* = 0.0192; *CR* = 0.0154 < 0.10 OK

**Table 4:** Priority vector and consistency analysis of SOCEM matrix assessing the social indicators

	CA	PA	MA	Priority vector
CA	1	1.2203	0.7025	0.3105
PA	0.8195	1	0.6504	0.2650
MA	1.4235	1.5375	1	0.4244

*λ*_max_ = 3.0017; *CI* = 0.0008; *CR* = 0.0016 < 0.05 OK

**Table 5:** Priority vector and consistency analysis of ECOEM matrix assessing the economic indicators

	*EL*	*IC*	*MC*	*OP*	Priority vector
EL	1	0.6934	0.2295	0.2131	0.0857
IC	1.4422	1	0.3364	0.3451	0.1272
MC	4.3580	2.9730	1	2.1017	0.4647
OP	4.6922	2.8976	0.4758	1	0.3224

*λ*_max_ = 4.0756; CI = 0.0252; *CR* = 0.0283 < 0.09 OK

$$CR=\frac{CI}{RCI}$$

*CI* can be calculated as follows:

$$CI=\frac{{\lambda }_{\text{max}}-n}{n-1}$$

where *λ*_max_ is the maximum eigenvalue of the elicitation matrix, and *n* is the matrix order. This elicitation matrix order is the number of TBL dimensions or drivers under study.

## Results

The eigenvector method has been applied to the four elicitation matrices: TBLEM, ENVEM, SOCEM, and ECOEM. The principal eigenvector is used to derive priorities from a pairwise comparison matrix. This matrix often contains judgments that are not perfectly consistent. The eigenvector method in AHP ensures that the derived priorities are consistent, unique, and mathematically sound, making it a reliable tool for decision-making. As shown in Table 2, the environmental dimension and the social dimension have obtained the highest weights, 46.75% and 37.34%, respectively. The high weights assigned to the environmental and social dimensions likely reflect their critical importance in the context being analyzed. Environmental dimension considers sustainability, stakeholder expectations which increasingly demand environmentally responsible practices, and regulatory compliance, whose high weighting ensures compliance and minimizes legal risks. Social dimension includes human rights and labor practices, social license to operate; and diversity and inclusion, emphasizing social aspects can help foster a diverse and inclusive workplace, which can enhance innovation and employee satisfaction. The ENVEM elicitation matrix, shown in Table 3, compares six drivers to evaluate the environmental dimension of the proposed water resources. The first driver is the synergistic use of infrastructures (*SU*), which measures the percentage shared by the different infrastructures necessary to convey water from the water resources to the wetland. Use of infrastructure for more than one purpose improves sustainability. As shown in Table 3, the priority obtained for the *SU* driver is 6.26%. The water resources’ location driver (*RL*) accounts for the location of the water resources with respect to the wetland. The score for this driver is higher as the distance is shorter. Long distances have a greater environmental impact than short ones. The priority of *RL* is 6.43%, and therefore similar to the *SU* one. The wastewater reuse driver (*WR*) and the energy consumption driver (*EC*) have a weight of 12.92% and 14.81%, respectively. Reusing wastewater and consuming less energy are favorable factors for reducing the impact on the environment. Landscape degradation (LD) with 23.81% and impact on the state of water resources (IM) with 35.76% have been ranked as the most significant environmental drivers. The construction of new infrastructures and the reduction of the amount of water in the reservoirs or donor aquifers are therefore key parameters for the environmental dimension. Finally, Table 6 shows the environmental dimension evaluation for the ten water resources under study. The evaluation of the environmental dimension is obtained adding the values of each driver multiplied by its corresponding priority. As shown, the top rated environmental water resources are VIRP (91.90%) and MARP (88.77%), therefore wastewater treatment plants are identified as the most sustainable resource for TDNP.

**Table 6:** Environmental dimension bottom line rating for the water resources under assessment

Water resource	*SU* (0.0626)	*RL* (0.0643)	*WR* (0.1293)	*EC* (0.1481)	*LD* (0.2381)	*IM* (0.3576)	ENBL priority
VICR	0.2512	0.8424	0.0000	0.0000	0.5364	0.0000	0.1976
GASR	1.0000	0.8850	0.0000	0.9424	0.6020	0.7372	0.6660
TOAR	1.0000	0.6854	0.0000	1.0000	0.6020	0.8464	0.7008
PVAR	0.5371	0.6486	0.0000	1.0000	0.3402	0.1225	0.3482
PARR	0.0697	0.6300	0.0000	1.0000	0.0000	0.8998	0.5148
TASE	1.0000	0.0000	0.0000	1.0000	1.0000	0.9920	0.8036
VILW	0.4127	0.9581	0.0000	0.1112	0.8901	1.0000	0.6734
FUFW	0.0000	0.9284	0.0000	0.5132	0.7853	1.0000	0.6803
MARP	0.9378	0.8134	1.0000	1.0000	0.5949	1.0000	0.8877
VIRP	0.3698	0.9576	1.0000	1.0000	0.8367	1.0000	0.9190

Three drivers have been applied to evaluate the social dimension, as shown in Table 4. The community acceptance driver (CA) evaluates the response of local residents. The priority obtained for this driver is 31.05%. The political acceptance (PA) is the second component of social acceptance with a weight of 26.50%. For this driver, political acceptance is evaluated ranking the level of alignment of the alternative with the water plan (BOE 2016). This decree approved the revision of the hydrological plans for several river basin districts in Spain. The decree aimed to update the management plans to ensure sustainable water use, improve water quality, and protect aquatic ecosystems. It incorporated the requirements of the European Union’s Water Framework Directive, focusing on environmental sustainability, economic rationality, and social transparency. The market acceptance driver (MA), accounting for the possible impact on irrigation, has a priority of 42.44%. Finally, applying the three social acceptance drivers, the best ranked water resources, as shown in Table 7 are TASE, VILW, and FUFW, while VICR is the worst rated for the social dimension.

**Table 7:** Social dimension bottom line rating for the water resources under assessment

Water resources	CA (0.3105)	PA (0.2650)	MA (0.4244)	SOBL priority
VICR	1.0000	0.2288	0.0000	0.3712
GASR	0.3194	0.0754	0.3117	0.2515
TOAR	1.0000	0.0754	0.8334	0.6842
PVAR	0.0749	0.0754	1.0000	0.4677
PARR	1.0000	0.0754	0.8334	0.6842
TASE	1.0000	1.0000	1.0000	1.0000
VILW	1.0000	1.0000	1.0000	1.0000
FUFW	1.0000	1.0000	1.0000	1.0000
MARP	1.0000	0.5441	0.1143	0.5033
VIRP	1.0000	0.5441	0.1143	0.5033

Table 5 shows the four drivers analyzing the economic dimension of TBL. The cost of expropriation of land and the cost of infrastructure have relatively low weights, 8.56 and 12.71%. This is because they are initial costs. On the other hand, the maintenance and opportunity costs are associated with the operation of the infrastructure and the volume of water diverted to the wetland. Their priorities are higher because they are periodic costs, associated with the operation of the infrastructure throughout its life cycle. As shown in Table 8, the best rated water resources for the economic dimension are VIRP (79.09%), FUFW (62.73%), VILW (59.17%), and TOAR (58.10%), while the worst ranked are PARR (13.13%), VICR (35.40%), and PVAR (37.19%).

**Table 8:** Economic dimension bottom line rating for the water resources under assessment

Water resources	*EL* (0.0857)	*IC* (0.1272)	*MC* (0.4647)	*OP* (0.3224)	ECBL priority
VICR	0.3729	0.5798	0.0000	0.7702	0.3540
GASR	0.5398	0.6967	0.6148	0.0000	0.4206
TOAR	0.5398	0.0000	0.5970	0.7982	0.5810
PVAR	0.1497	0.0904	0.0542	1.0000	0.3719
PARR	0.0000	0.0255	0.0384	0.3418	0.1313
TASE	1.0000	1.0000	0.3536	0.4323	0.5166
VILW	0.8946	0.8927	0.5129	0.5061	0.5917
FUFW	0.8109	0.8106	0.6274	0.5061	0.6273
MARP	0.3530	0.4796	0.5304	0.5061	0.5009
VIRP	0.9189	0.8785	0.7950	0.7165	0.7909

Finally, considering together the three dimensions of TBL, as shown in Table 9, TASE is the best option with a rating of 83.13%, closely followed by FUFW (79.12%), VILW (78.24%), VIRP (74.34%), MARP (68.26%), and TOAR (67.56%). Except for the TOAR case, the reservoirs have low ratings due to the economic dimension: PARR (51.71%), GASR (47.22%), PVAR (39.66%), and VICR (28.73%). TASE is the highest rated water source because it is already constructed, and no pumping is needed to convey water to the Tablas de Daimiel wetland. Additionally, the impact on water resources is negligible because the volume of water derived is very small compared to the capacity of Entrepeñas and Buendía reservoirs from where the water is provided. Moreover, this pipeline has also been designed to supply fresh water to the population of the vicinity. And therefore, SU driver is also top ranked for this water source. However, the *WR* and *RL* drivers are low rated for TASE because water is coming from reservoirs, and the distance from the water source to the Tablas de Daimiel wetland is the largest one. Social drivers are also top rated for TASE, as there is no opposition from farmers or population, and TASE is included in the Water Plan. The economic drivers obtained for TASE are top ranked for *EL* and *IC* drivers because it is already constructed, but MC and OP drivers are low rated.

**Table 9:** Triple bottom line rating for the water resources under assessment

Water resources	ENBL (0.4676)	SOBL (0.3734)	ECBL (0.1590)	TBL priority
VICR	0.1976	0.3712	0.3540	0.2873
GASR	0.6660	0.2515	0.4206	0.4722
TOAR	0.7008	0.6842	0.5810	0.6756
PVAR	0.3482	0.4677	0.3719	0.3966
PARR	0.5148	0.6842	0.1313	0.5171
TASE	0.8036	1.0000	0.5166	0.8313
VILW	0.6734	1.0000	0.5917	0.7824
FUFW	0.6803	1.0000	0.6273	0.7912
MARP	0.8877	0.5033	0.5009	0.6826
VIRP	0.9190	0.5033	0.7909	0.7434

## Conclusions

**Fig. 3:**
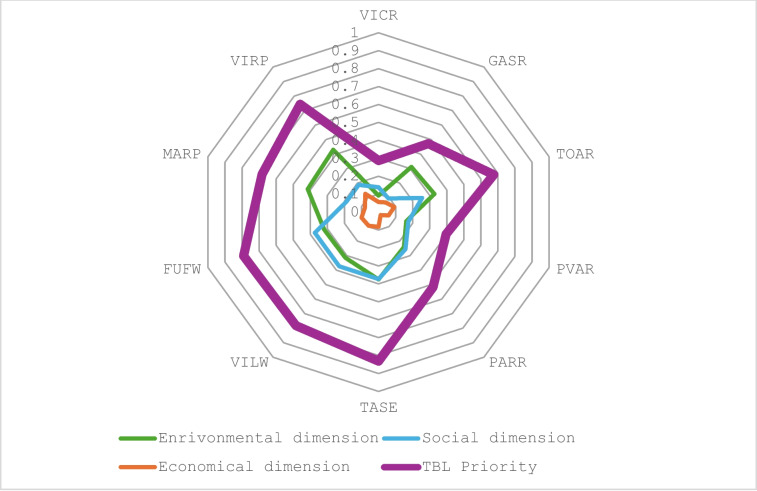
Triple bottom line rating for the ten water resources under assessment

Wetland benefits are widely known: water depuration, flood abatement, erosion protection, and water and food supplying to population, in addition to being important biodiversity reservoirs on the Planet. In many cases, hydrological restoration of wetlands needs supplying water from external resources, especially in arid and semiarid regions of the Mediterranean areas impacted by climate change. The holistic index has proven to be a flexible and efficient hybrid technique for prioritizing the water resources for the TDNP restoration. The water management proposal must be sustainable, minimizing the environmental and social impacts. To this end, the drivers used for the elicitation have been structured on the triple bottom line to account the environmental, the social, and the economic dimensions (Fig. 3). An elicitation technique using AHP ratings with drivers has been successfully applied to identify water resources options for the hydrological restoration of the Tablas de Daimiel wetland. Ten available water resources have been analyzed: groundwater, reservoirs, interbasin water transfer, and recycled water resources. However, the use of wastewaters will be sustainable only if they are adequately treated to prevent negative pollution impacts. A future research direction to restore the TDNP could be to identify how water extraction could be reduced to restore the aquifer, e.g., by reducing water intake for agriculture through modification of irrigation methods or selection of crops better adapted to the current climate using the same approach. The ethical considerations revolve around the equitable distribution of resources and the protection of environmental ecosystems. Fortunately, our hybrid method allows adding new water resources without reviewing the hierarchical framework, avoiding the repetition of all the elicitation process. Because the devised drivers guarantee traceability and transparency in decision making, we believe our approach is a promising management framework to support policy makers in wetland conservation.

## Data Availability

All the data generated or analyzed during this study are included in this published article.
